# Obesity, Nutrition and the Multiple Sclerosis Risk in Adolescents

**DOI:** 10.3390/brainsci16030283

**Published:** 2026-02-28

**Authors:** Marta Giovengo, Margherita Rosa, Claudia Mandato

**Affiliations:** 1 Department of Medicine and Surgery, Scuola Medica Salernitana, University of Salerno, 84084 Salerno, Italy; 2 Division of Chronic Diseases, Hepatology, and Nutrition, Santobono-Pausilipon Pediatric Hospital, 80129 Naples, Italy

**Keywords:** multiple sclerosis, pediatric obesity, gut microbiota, gut–liver–brain axis, mediterranean diet, GLP-1 agonists, neuroinflammation

## Abstract

**Highlights:**

**What are the main findings?**

- Multiple Sclerosis (MS) involves a distinct prodromal phase. Obesity is an independent risk factor, inducing metabolic stress and epigenetic reprogramming that establish a pro-inflammatory environment that lowers the threshold for autoimmune activation.
- The pathogenesis of MS involves a dysfunctional gut–liver–brain axis where diet-induced dysbiosis and secondary bile acid modification trigger systemic inflammation, leading to blood–brain barrier disruption and sustained neuroinflammation.

**What are the implications of the main findings?**

- The Mediterranean Diet serves as a biologically active determinant of immune regulation rather than a mere lifestyle factor, with higher adherence significantly associated with reduced MS risk and improved clinical outcomes.
- Metabolic interventions, including GLP-1 receptor agonists and bile acid modulation, represent promising therapeutic strategies that can provide dual benefits by managing obesity while exerting direct neuroprotective effects.

**Abstract:**

Multiple Sclerosis (MS) is a chronic, autoimmune neurological disease resulting from the interplay between genetic susceptibility and environmental factors. In recent decades, the rising incidence of MS, particularly pediatric-onset forms, has paralleled the global obesity pandemic. This article explores the causal link between pediatric obesity, systemic inflammation, and neuroinflammation, with a specific focus on the microbiota–gut–liver–brain axis. We analyze how nutritional habits can play a pivotal role by inducing dysbiosis, with alteration in microbiota-driven metabolites, and leaky gut related abnormalities—which may trigger blood–brain barrier (BBB) disruption and microglial activation—or by acting as a protective factor, such as through the Mediterranean Diet (MD). Furthermore, we evaluate the emerging therapeutic perspectives offered by Glucagon-Like Peptide-1 (GLP-1) agonists, which may offer dual benefits in weight management and immune modulation.

## 1. Introduction

Pediatric obesity, defined as Body Mass Index (BMI) ≥ 95th percentile for age and sex, has become one of the most significant public health issues worldwide. Indeed, the global prevalence of overweight and obesity in children and adolescents is increasing at an alarming rate. Forecasting studies indicate that obesity rates will continue to rise dramatically across almost all Countries by 2050 [1]. Running parallel to this metabolic epidemic, the epidemiological landscape of Multiple Sclerosis (MS) has shifted significantly in recent decades, with increasing prevalence of cases presenting during childhood and adolescence [2]. The connection between these two trends is far from coincidental. It is now well-established that obesity during early adolescence effectively doubles the risk of developing MS [3,4]. Multiple population-based studies have confirmed the relationship between high BMI in adolescence and young adulthood (ages 14–24) and subsequent MS risk [3,5]. Perhaps most striking, large-scale prospective cohort studies have demonstrated that high BMI in childhood is a significant independent risk factor for MS. In these cohorts, hazard ratios exceeding 2.0 in obese cohorts compared to the general population [4]. The pathogenesis of MS involves a complex interplay between genetic susceptibility (e.g., HLA-DRB1*15), immune dysregulation, and other environmental exposures (including Vitamin D deficiency, smoking, diet and *Epstein–Barr virus* infection). However, the risk associated with obesity remains significant even after adjusting for these factors. Furthermore, this risk is strongly dependent on the severity of the obesity [6]. This observation suggests that obesity-related metabolic dysfunction may play a central role in MS pathogenesis, expanding possible preventive targeted strategies.

**Figure 1:**
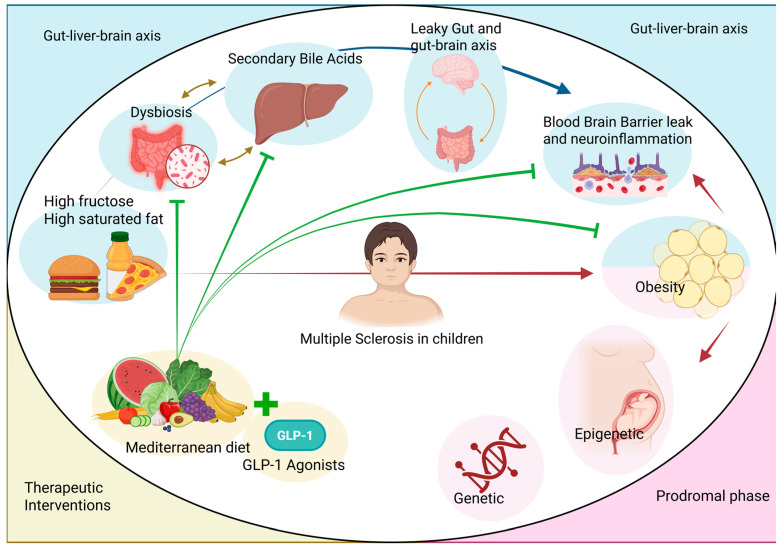
Pathogenesis of Multiple Sclerosis as a complex crosstalk between nutrition, the gut–liver–brain axis, obesity, and epigenetic programming. Pathological Axis (Top): The upper panel illustrates how diets high in fructose and saturated fats induce intestinal dysbiosis, which in turn alters hepatic metabolism and the production of secondary bile acids. This cascade results in “leaky gut” and increased permeability of the blood–brain barrier, allowing pro-inflammatory triggers to reach the central nervous system. Prodromal Phase (Bottom-Right): This section depicts the convergence of genetic susceptibility and epigenetic modifications. When exacerbated by obesity, these factors create a self-sustaining loop of systemic inflammation that precedes clinical onset. Therapeutic Interventions (Bottom-Left): The Mediterranean Diet and GLP-1 receptor agonists are highlighted as critical modulators. These interventions exert inhibitory signals (represented by solid green T-bars) that directly counteract Western diet-induced dysbiosis, secondary bile acid signaling, obesity and neuroinflammation. By modulating these pathways, these therapies potentially prevent or mitigate the transition from subclinical inflammation to overt neuroinflammation and demyelination.

To better visualize these complex interactions, Figure 1 provides a comprehensive schematic of the microbiota–gut–liver–brain axis, illustrating how metabolic stress, dysbiosis, and obesity-driven systemic inflammation converge to trigger neuroinflammation in the pediatric population, while highlighting the potential role of targeted therapeutic interventions.

## 2. Methodologies

This article is a narrative review. A comprehensive literature search was conducted using PubMed, Scopus, Web of Science, and Google Scholar databases to identify relevant studies published primarily in the last 10 years, up to January 2026. The search strategy combined the following keywords: ‘Multiple Sclerosis’, ‘Pediatric Obesity’, ‘Gut Microbiota’, ‘Gut–Liver Axis’, ‘Mediterranean Diet’, and ‘GLP-1 Agonists’.

While we prioritized systematic reviews, meta-analyses, and clinical trials published in the last decade to ensure up-to-date evidence, seminal historical papers and highly relevant mechanistic studies were also included to provide necessary context and depth to the discussion. The selection of articles was guided by their specific relevance to the pathophysiological link between metabolic dysfunction and neuroinflammation. Animal/preclinical studies were selected when they were considered particularly promising and noteworthy in relation to the aim of the review and future therapeutic perspectives. Regarding clinical studies, we attempted to select predominantly pediatric or mixed (pediatric and adult) studies in order to maintain a specific focus on the pediatric age group. Some studies conducted in adults are also cited because they were considered particularly relevant for advancing scientific knowledge. A summary of the search strategy and the databases consulted is provided in Table 1.

Finally, it should be noted that as a narrative review, this work possesses inherent limitations, such as the potential for selection bias and the absence of a systematic, reproducible protocol typical of systematic reviews. However, every effort was made to ensure a balanced and critical synthesis of the available literature.

## 3. Prodromal Phase and Epigenetic Programming

Growing evidence indicates that the pathogenic process of MS begins long before the appearance of overt neurological manifestations. Recent population-based studies have identified a distinct “prodromal phase” in pediatric MS. This period can last up to five years before a formal diagnosis, and affected individuals exhibit non-specific clinical symptoms, including visual disturbances, recurrent respiratory infections, and fatigue. These findings suggest that immune dysregulation and central nervous system (CNS) vulnerability may already be established during early childhood, and even before, long preceding the onset of clinical disease [7]. During the prodromal phase, the interplay between environmental triggers and genetic susceptibility is mediated by a complex epigenetic landscape. This phase is not merely a clinical latency but a period of active biological reprogramming where environmental signals are translated into gene expression changes [8].

### 3.1. Epigenetic Mechanisms in Multiple Sclerosis

MS has a known genetic component, evidenced by hundreds of single-nucleotide polymorphisms (SNPs) and high prevalence of certain HLA haplotypes (*DRB11501*, *DQB10602*) on chromosome 6p21. However, the concordance rate among monozygotic twins is only 20–30%. This indicates that genetic predisposition alone is insufficient for the MS development [2]. These observations highlight the role of epigenetic modifications in regulating gene activation and repression. Epigenetic mechanisms act as critical molecular interfaces between environmental exposures and gene regulation, modulating immune function without altering the underlying DNA sequence. These mechanisms involve methylation, acetylation, phosphorylation, ribosylation, and ubiquitination of DNA or associated histone proteins and non-coding RNA regulation.

MicroRNA (miRNA)-mediated gene silencing represents a key post-transcriptional regulatory process. miRNAs are small non-coding RNAs (21–23 nucleotides) that are incorporated into the RNA-induced silencing complex (RISC) to regulate target mRNAs. miRNA–mRNA interactions influence cellular processes including differentiation, proliferation, and apoptosis. In MS, several miRNAs are overexpressed and upregulated in active MS lesions and play a role in immune regulation, particularly in Th17 cell differentiation [9].

Integrated analysis of miRNA and mRNA in pediatric patients has revealed unique pathogenic signatures. These signatures distinguish pediatric from adult forms, highlighting the early involvement of pathways related to cell cycle control and immune activation [10].

miRNAs modulate the permeability of both the intestinal barrier and blood–brain barrier (BBB), thereby influencing the complex crosstalk between the gut microbiota, the immune system, and the CNS. In particular, preclinical and clinical studies in both animal models and humans have shown that miRNAs regulate the expression of key genes involved in gut barrier integrity and modulate the interaction between gut microbiota and the host immune system [11,12]. For instance, in pediatric patients the upregulation of miR-155 promotes a Th17-mediated pro-inflammatory environment while impairing Treg-mediated tolerance, a critical step in the transition from subclinical inflammation to overt demyelination [11,13,14]. Furthermore, miRNAs have also emerged as critical regulators of BBB integrity by directly targeting endothelial tight junctions or by regulating endothelial cell survival, inflammatory pathways, and apoptosis [12]. These processes are further complicated by the existence of miRNA-transcription factor co-regulatory networks, which create self-sustaining inflammatory loops within the microbiota–gut–brain axis [12,15].

Taken together, accumulating evidence positions miRNA dysregulation as a central epigenetic hub integrating environmental exposures, microbial-derived signals, and host immune–metabolic responses along the gut–liver–brain axis. Through the coordinated modulation of intestinal permeability, hepatic immune surveillance, and blood–brain barrier integrity, altered miRNA networks may orchestrate the transition from peripheral immune imbalance to compartmentalized neuroinflammation, thereby contributing to both the initiation and chronic propagation of MS pathology.

### 3.2. Pregnancy and Epigenetic Programming

Emerging evidence from metabolic disorders, including obesity and diabetes, indicates that such epigenetic alterations may arise as early as the gametes stage and immediately after fertilization [16].

Notably, fetal DNA methylation patterns are influenced by maternal diet and metabolic status: maternal metabolic dysfunction and suboptimal nutritional patterns before and during pregnancy have been associated with epigenetic modifications in genes involved in immune regulation, adipogenesis, and neuroinflammatory pathways. As a result, they may predispose offspring to metabolic and immune-mediated disorders, including MS [17].

Moreover, a recent and relevant study reported that being born large for gestational age (LGA) and prenatal exposure to maternal diabetes were associated with an increased risk of adult-onset MS. Conversely, being born small for gestational age (SGA) was associated with a reduced risk. These findings suggest that disease susceptibility may originate as early as the prenatal period, highlighting the potential role of intrauterine metabolic programming in shaping long-term MS risk [18].

These transgenerational epigenetic effects provide a plausible biological framework linking maternal lifestyle and nutrition to long-term neuroimmune vulnerability in the offspring [16].

### 3.3. Obesity and Nutritional Factors Influence

In the postnatal period, dietary factors deserve special attention. Many of the beneficial effects of bioactive vegetable compounds involve the modulation of specific transcription modulators, such as HDAC proteins. Inhibiting these proteins leads to increased global acetylation of histones H3 and H4, thereby modulating the expression of multiple genes. Cruciferous vegetables, such as broccoli, rich in sulforaphane, can alter HDAC activity in circulating human cells. Similarly, butyrate, produced by the fermentation of dietary fiber by the gut microbiota, and S-allyl-mercaptocysteine, derived from garlic metabolism, also exhibit HDAC-inhibitory activity. These rapid and transient epigenetic changes induced by dietary molecules, shown in preclinical studies and preliminary translational and human data, are proposed to have biological significance, potentially modulating gene expression in ways that influence disease susceptibility [9].

Obese adolescents exhibit a prodromal pro-inflammatory environment. This state is characterized by altered adipokine profiles, such as elevated levels of Leptin and FABP4. These changes lower the threshold for autoimmune activation [19]. This environment is further shaped by specific miRNAs—such as miR-16-5p, miR-124-3p, miR-103a-3p, and miR-107—that modulate genes involved in inflammation, lipid metabolism, and brain development. Together, these factors drive the immune and neuroinflammatory processes essential to MS pathogenesis [20].

This metabolic stress is reflected in the circulating extracellular vesicle (EV) miRNA signatures, where specific markers like miR-34a-5p and miR-140-5p—already linked to brain atrophy and disease activity in early MS—serve as systemic indicators of obesity-driven immune dysregulation [21,22].

### 3.4. Future Implications

The evolving understanding of the epigenetic landscape in MS paves the way for novel therapeutic strategies. Recent breakthroughs have identified a specific subset of epigenetically regulated “memory astrocytes”. These cells contribute significantly to CNS pathology in experimental autoimmune encephalomyelitis in mice (EAE) and potentially in MS lesions in human cells in vitro. This astrocyte population displays enhanced pro-inflammatory responses and is regulated by histone acetylation, which controls chromatin accessibility. The number of these memory astrocytes is increased in both EAE models and chronic MS lesions, and their genetic or pharmacological inactivation may ameliorate disease severity, highlighting a potential therapeutic target in neuroinflammatory disorders [23]. These findings are particularly relevant in the context of pediatric MS. Since the pediatric brain is in a critical stage of development characterized by high neuroplasticity, it may be vulnerable to the “imprinting” of such epigenetic memories. Early intervention targeting these memory astrocytes could therefore prevent the consolidation of self-sustaining inflammatory loops, offering a crucial window of opportunity to alter the long-term disease trajectory in younger patients more effectively than in adults.

Parallel to astrocyte memory, oligodendroglial cells from both mice and humans display distinct chromatin accessibility patterns. Evidence indicates that oligodendrocytes maintain a “primed” immune chromatin state. This state persists even under non-disease conditions, suggesting an epigenetic memory of prior inflammatory events, highlighting oligodendroglial cells as potential targets for immunomodulatory therapies in MS [24].

Furthermore, another recent research has demonstrated that MS oligodendrocytes lesion-resident are epigenetically silenced. The identification of a small-molecule epigenetic silencing inhibitor (ESI1) offers a promising therapeutic avenue; ESI1 reactivates myelin gene expression, promotes remyelination in animal models and human organoids, and improves neurological function recovery in mice, suggesting a promising strategy for demyelinating diseases [25].

Moreover, evidence suggests that neuronal cell death mechanisms occurring during neuroinflammation are tightly linked to epigenetic regulation. Recent human proof-of-concept studies indicate that the analysis of methylation profiles of neuron-derived cell-free DNA (cfDNA) in cerebrospinal fluid or plasma may provide novel, non-invasive diagnostic and prognostic tools for tracking neurodegenerative progression in MS patients [26].

## 4. Pathophysiological Mechanisms: The Microbiota–Gut–Liver–Brain Axis

The “Gut–Liver–Brain Axis” is a bidirectional signaling pathway linking intestinal and liver driven metabolites, immune system to CNS immune regulation. Alteration in this pathway is emerging as a possible pathomechanism of MS in obese patients.

In this paradigm, excess adipose tissue functions as an active endocrine organ. It engages in dynamic cross-talk with skeletal muscle, liver, cardiovascular system, and brain, exerting systemic effects. In individuals with obesity, adipose tissue exhibits profound hormonal and metabolic dysregulation. This leads to an abnormal adipokine profile characterized by increased secretion of pro-inflammatory mediators, including leptin, tumor necrosis factor-α (TNF-α), resistin, and retinol-binding protein-4 (RBP-4), alongside a reduction in anti-inflammatory adipokines.

Beyond adipocyte-derived cytokines, adipose tissue expansion is associated with increased infiltration of macrophages, lymphocytes, and other immunologically active cells. Together, these cells sustain a state of chronic low-grade inflammation. This condition, commonly referred to as metabolic inflammation or metainflammation, is further exacerbated by obesity-related disturbances in endocrine regulation, including dysregulation of the growth hormone–insulin-like growth factor-1 (GH–IGF-1) axis and increased cortisol bioavailability [27].

### 4.1. Dietary Fructose and Metabolic Toxicity

Nutritional habits play a pivotal role in modulating the metabolic risk [16]. In particular, Western dietary patterns are characterized by excessive added sugar consumption, as reported in a recent consensus statement [28]. Possible added sugars are both glucose and fructose. Fructose-rich diets are particularly harmful because, unlike glucose, fructose undergoes unregulated hepatic metabolism through multiple mechanisms, including the promotion of de novo lipogenesis, visceral fat accumulation, and increased endoplasmic reticulum (ER) stress. This promotes hepatosteatosis and contributes to the development of Metabolic Dysfunction-Associated Steatotic Liver Disease (MASLD). In addition, fructose-induced MASLD is modulated by the intestinal epithelial barrier via the gut–liver axis. Excessive fructose consumption leads to gut barrier deterioration, dysbiosis, low-grade intestinal inflammation, and endotoxemia, which will be discussed in more detail below [29]. Overall, this phenomenon has been termed “Sweet Death,” reflecting fructose’s role as a metabolic toxin, in particular generating oxidative stress and mitochondrial dysfunction and contributing to a pro-inflammatory systemic environment [30]. Therefore, the inflamed liver releases cytokines which, together with signals from adipose tissue, may increase BBB permeability, facilitating immune cell infiltration into the CNS [31].

### 4.2. Gut Dysbiosis and Bile Acids Metabolism

Adding a further layer of complexity, high-sugar and high-saturated-fat diets induce profound alterations in gut microbiota composition [32,33]. Under physiological conditions, the human gut microbiota—dominated by the Firmicutes and Bacteroidetes phyla—maintains a dynamic equilibrium with the host. However, genetic and environmental influences may disrupt this balance, leading to gut dysbiosis, characterized by reduced microbial diversity, shifts in the Firmicutes/Bacteroidetes ratio, and decreased production of protective short-chain fatty acids (SCFAs), such as butyrate [32].

A critical consequence of dysbiosis is the impairment of bile acid (BA) metabolism, a key component of the gut–liver axis, which operates as a bidirectional host-defense system in which BAs function as pivotal signaling molecules. Primary BAs are synthesized in the liver and secreted into duodenum. There, they are converted by the gut microbiota into secondary BAs through bacterial deconjugation mediated by enzymes such as bile salt hydrolases. These secondary BAs exert antimicrobial activity and act as potent ligands for receptors including the farnesoid X receptor (FXR) and the G protein–coupled bile acid receptor TGR5.

Activation of FXR plays a central role in regulating bile acid homeostasis, lipid and glucose metabolism, intestinal barrier integrity, and immune responses [34]. TGR5 signaling contributes to anti-inflammatory effects in immune cells. These receptors are expressed in the CNS, influencing neuroinflammatory responses, microglial activation, and neuroendocrine signaling. FXR is upregulated during neuroinflammation and appears to contribute to neurotransmitter homeostasis and behavioral regulation [35]; TGR5 is expressed in microglia and astrocytes; its stimulation attenuates the release of pro-inflammatory cytokines and reduces neuroinflammation [36]. Preclinical and clinical studies show that dysbiosis-associated alterations in the BAs pool disrupt FXR- and TGR5-mediated signaling. This disruption leads to impaired anti-inflammatory responses in macrophages and T cells and promoting T-cell differentiation towards pro-inflammatory phenotypes, such as Th17 cells [37].

Altered bile acid profiles have been observed in both serum and cerebrospinal fluid of patients with MS and correlate with disease activity and immune dysregulation. Specifically, serum bile acid profiles are significantly altered in children and adolescents with MS, exhibiting reduced levels compared to healthy controls [36]. This dysregulation reflects a profound dysfunction of the gut–liver–brain axis. Beyond that, the systemic biochemical profile of the liver–brain axis can be further characterized by enzymatic markers of oxidative stress. In this context, gamma-glutamyltransferase (GGT)—a key enzyme in glutathione metabolism—emerge as a marker of disease activity. Elevated serum GGT activity has been associated with MS since the late 1970s, reflecting systemic metabolic disturbances [38]. More recently, GGT has been recognized as a sensitive marker of the oxidative stress that drives BBB damage and neuroinflammatory progression. Clinical evidence indicates that both MS and neuromyelitis optica (NMO) adult patients exhibit significantly higher serum GGT levels compared to healthy controls, with GGT levels correlating positively with disability scores (EDSS) and lesion extension [39].

Collectively, these mechanisms provide a biological framework through which diet-induced dysbiosis and bile acid dysregulation may exacerbate immune dysfunction and contribute to the pathogenesis and progression of MS [37,40].

### 4.3. Intestinal Permeability and Neuroinflammation

One of the key consequences of diet-induced dysbiosis is the impairment of intestinal epithelial barrier integrity. Experimental models have shown that a high-fat diet is associated with a marked reduction in tight junction (TJ) proteins, which normally protect against the translocation of pathogen-associated molecular patterns (PAMPs). BAs pool has also been shown to regulate the formation and maintenance of the intestinal mucus layer and to exert a pivotal role in preserving epithelial barrier integrity through FXR signaling. This process is part of a complex and tightly regulated network involving diet, gut microbiota, host nuclear receptors, and intestinal barrier function [41]. The loss of TJ integrity results in increased intestinal permeability, commonly referred to as a “leaky gut”. This condition facilitates the translocation of Lipopolysaccharides (LPSs) into the systemic circulation. Once in circulation, LPS activates toll-like receptors (TLRs), particularly TLR4. This activation promotes the secretion of pro-inflammatory cytokines and contributing to hepatic inflammation [30]. Beyond the liver, circulating LPS can also activate TLR4 expressed on innate immune cells and CNS microglia, leading to increased BBB permeability and sustained neuroinflammation. This mechanism provides biological plausibility for the concept of “smoldering inflammation,” a chronic, low-grade inflammatory state that may be present even during the early stages of MS [2].

## 5. Therapeutic Interventions

Given that metabolic drivers appear to fuel neuroinflammation, restoring metabolic homeostasis represents a viable and attractive therapeutic target.

### 5.1. Nutritional Interventions: The Mediterranean Diet

While the Western diet is known to promote systemic and intestinal inflammation, the Mediterranean Diet (MD) has demonstrated a significant capacity to modulate the gut–immune axis in pediatric MS. Characterized by high intakes of dietary fiber, polyphenols, and monounsaturated fatty acids, the MD favors the expansion of butyrate-producing bacterial taxa, including members of the Ruminococcaceae family, and contributes to the restoration of intestinal barrier integrity and immune homeostasis [42]. Through these mechanisms, the MD exerts indirect immunomodulatory effects by reducing gut permeability, limiting endotoxin translocation, and promoting regulatory immune pathways. Moreover, as discussed in the previous section, distinct dietary regimens induce specific alterations in bile acid profiles, which are associated with divergent host physiological responses. Diet is a key driver of variability in microbial and bile acid metabolism in murine models and in limited human studies, underscoring the need for further human-centered studies to validate and extend current findings [41]. Recent pediatric case–control evidence further indicates that higher adherence to the MD is associated with a substantially lower likelihood of MS. Notably, diet explains a greater proportion of gut microbiota variation than disease status itself and specific microbial taxa appear to mediate this protective association [42]. The clinical relevance of these biological effects is supported by observational and interventional studies. In both pediatric and adult MS populations, higher adherence to the MD has consistently been associated with lower disease severity scores, improved clinical outcomes, reduced relapse risk, slower disability progression, and enhanced quality of life [43,44]. Of particular interest, a controlled dietary intervention study in Iranian adults with relapsing–remitting MS demonstrated that even a modified MD significantly reduced fatigue severity over a six-month period, whereas traditional dietary patterns failed to confer comparable benefits [45].

In a recent pediatric MS cohort, a higher alternative Mediterranean diet score (aMED) score and higher intakes of fiber (g/day), and iron (mg/day) were each associated with a lower likelihood of having MS. Particularly, a 1-point increase in the aMED score was associated with 34% lower odds of MS. Furthermore, MS cases showed an enrichment of the archaeon *Methanobrevibacter* and of *Eggerthella*, and the presence of *Methanobrevibacter* was associated with a markedly increased likelihood of MS. In contrast, MS cases were characterized by a depletion of the *Clostridiales vadinBB60* group and the *Ruminococcaceae NK4A214* group, the latter belonging to a family known for SCFAs production. Interestingly, statistical data indicate that diet mainly explains inter-individual gut microbiota variation [42]. These findings reinforce the concept that dietary quality is not merely an adjunctive lifestyle factor but a biologically active determinant of immune regulation in MS. Accordingly, comprehensive reviews of nutritional interventions in MS converge on the conclusion that diet composition plays a pivotal role in influencing disease trajectory and symptom burden [46].

It is important to note that heterogeneity in microbiome studies—arising from differences in sequencing methods, sample handling, bioinformatic pipelines, and cohort characteristics—can limit the comparability and generalizability of findings. Additionally, lifestyle factors such as physical activity, sunlight exposure or serum vitamin D levels, sleep patterns, medication use, dietary changes after symptom onset or diagnosis, and socio-economic conditions may further modulate gut microbiota composition and immune responses, potentially confounding diet–microbiome associations. Future studies with standardized methodologies and comprehensive data on daily lifestyle factors are warranted to clarify causal relationships and enhance translational relevance.

### 5.2. Pharmacological Interventions: GLP-1 Receptor Agonists

Beyond nutritional interventions, Glucagon-Like Peptide-1 GLP-1 receptor agonists, such as semaglutide and liraglutide, currently used for obesity and type-2 diabetes, are emerging as potential drugs in MS. The rationale is multifactorial: preclinical studies show that GLP-1 agonists reduce oxidative stress, preserve axonal integrity, suppress microglial and astrocytic activation, and may even promote remyelination in experimental models [47,48]. GLP-1 receptors are expressed on microglia and astrocytes and their activation inhibits the NF-kB pathway and reduces oxidative stress. This suggests that these agents not only promote weight loss and improve glycemic control but may also exert direct neuroprotective effects [49]. Early clinical data in adult MS patients are encouraging, indicating that GLP-1 agonists are well-tolerated, provide expected metabolic benefits and improve fatigue in MS patients. This potentially offers a dual strategy to manage both the metabolic risk and neurodegenerative disease progression [50,51]. However, we must acknowledge important limitations: no randomized controlled trials in MS have been conducted to date to evaluate safety, both in adult and in pediatric age, and rigorous prospective studies are essential to establish disease-modifying efficacy.

### 5.3. Pharmacological Interventions: Emerging Therapeutic Role of BAs

Considering the emerging role of secondary BAs in the pathogenesis of MS, it is possible that future therapeutic strategies will focus on the modulation of these metabolites and their receptors. The therapeutic potential of BAs extends beyond their traditional role in hepatobiliary diseases, where agents like Ursodeoxycholic acid (UDCA) and its derivatives have long been established [52,53]. Indeed, studies reveal that MS patients exhibit significantly altered bile acid profiles in both serum and CSF, correlating with disease severity and immune dysfunction [54].

In animal models studies, tauroursodeoxycholic acid (TUDCA) demonstrates that BAs can attenuate neuroinflammation by inhibiting the pro-inflammatory polarization of microglia and astrocytes, thereby reducing axonal damage and demyelination [36,55]. The activation of TGR5 has been shown to modulate energy homeostasis and immune responses. This suggests that targeting BAs receptors could offer a novel strategy to promote neuroprotection and mitigate disease progression in MS [40,53]. Furthermore, established MS treatments, such as dimethyl fumarate, exert immunomodulatory effects by activating the nuclear factor erythroid-derived 2-related factor 2 (Nrf2) transcription factor. This activation upregulates antioxidant and cytoprotective gene expression. Interestingly, secondary BAs have also been described in the literature as potent modulators of the Nrf2 pathway [56], suggesting a shared mechanistic pathway for neuroprotection. Importantly, a recent double-blind, placebo-controlled trial was conducted in adult individuals with progressive MS. This study demonstrates that TUDCA supplementation is safe, well tolerated, and produces measurable effects on CD4+ T cells and the gut microbiota. This findings support further evaluation in larger and longer-term studies [57].

## 6. Conclusions

Evidence indicates that MS risk is not solely influenced by genetic predisposition and adolescent environmental exposures but may be partially programmed during critical developmental windows spanning preconception, gestation, and early childhood. The recognition of a pediatric prodromal phase, coupled with evidence of epigenetic and transgenerational influences, supports the concept that preventive strategies could focus on early-life periods, potentially even before conception, through optimization of maternal nutrition, metabolic health, and inflammatory status.

We suggest that metabolic dysfunction represents a potential driver of neuroinflammation in pediatric MS with the Gut–Liver–Brain axis as the pathological mechanism linking nutrition, microbiota, and neuroinflammation. Since obesity and poor dietary habits are modifiable risk factors, we propose that early adoption of healthy lifestyles, particularly MD, and consideration of targeted metabolic therapies such as GLP-1 agonists may represent promising strategies into standard clinical management of MS, especially in the pediatric population, to mitigate inflammation and improve long-term outcomes.

## Figures and Tables

**Table 1:** Search Strategy Summary.

Item	Description
Databases	PubMed, Scopus, Web of Science, Google Scholar
Timeframe	From January 2015 to January 2026
Keywords	Multiple Sclerosis; Pediatric Obesity; Gut–Liver–Brain Axis; Dysbiosis; Fructose; Bile Acids; GLP-1; Mediterranean Diet; Neuroinflammation
Inclusion Criteria	Full-text articles in English; promising animal/preclinical studies; Prospective cohort studies; Clinical trials involving pediatric population and relevant clinical trials on adult MS populations; Reviews involving pediatric and adult MS populations
Exclusion Criteria	Abstracts only; Case reports; Non-English publications

## Data Availability

No new data were created or analyzed in this study.

## Abbreviations

The following abbreviations are used in this manuscript:

aMED	alternative Mediterranean Diet score
BA	Bile Acid
BBB	Blood–Brain Barrier
BMI	Body Mass Index
CNS	Central Nervous System
FXR	Farnesoid X Receptor
GLP-1	Glucagon-Like Peptide-1
LPSs	Lipopolysaccharides
MASLD	Metabolic Dysfunction-Associated Steatotic Liver Disease
MD	Mediterranean Diet
MS	Multiple Sclerosis
NF-kB	Nuclear Factor kappa-light-chain-enhancer of activated B cells
SCFAs	Short-Chain Fatty Acids
TGR5	G-protein-coupled Receptor 5
Th17	T helper 17 cells
TLR4	Toll-Like Receptor 4
TNF-α	Tumor Necrosis Factor-alpha
